# Enhanced Weather-Based Index Insurance Design for Hedging Crop Yield Risk

**DOI:** 10.3389/fpls.2022.895183

**Published:** 2022-07-22

**Authors:** Yan Sun

**Affiliations:** School of Economics and Management, Institute of Disaster Prevention, Langfang, China

**Keywords:** weather index-based insurance, weather-yield model, basis risk, contract design, optimization approach

## Abstract

This study proposes an optimization-based weather-yield model to reduce the basis risk of weather-based index insurance. This weather-yield model helps us capture the growing season's monthly variation as it involves monthly explanatory weather indices. In addition, it can capture additional extreme weather effects by including extreme cooling or heating weather indices. This study presents an innovative machine learning framework incorporating optimization approaches to ensure the parsimony of weather index models and the accuracy of crop yield predictions, which can be integrated into the conventional policy design and pricing process. The advantages of this modeling approach and the effectiveness of weather index-based insurance based on this approach in reducing basis risk and revenue risk are demonstrated by applying county-level yield data for mid-season rice in the Anhui province, China.

## Introduction

According to the World Food Summit (1996), “food security exists when all people, at all times, have physical and economic access to sufficient, safe and nutritious food that meets their dietary needs and food preferences for an active and healthy life.” This widely accepted definition points to four dimensions of food security, i.e., food availability, food access, utilization, and stability. Agricultural insurance has been playing an important role in addressing some of these dimensions by reducing the vulnerability of the global food system to acute food shocks, and thereby contributing to food security's resilience and sustainability. However, finding an effective and sustainable risk management approach for agricultural producers, insurance providers, and the government has proven to be extremely challenging. The urgency of this problem is further aggravated by the estimation that food productivity needs to be increased by 70% to feed the world's growing population by 2050 (FAO, 2009) and the mounting concerns over possible changes in climate, which can lead to significant widespread agricultural losses.

Prompted by the critical role of agricultural insurance as a risk mitigation strategy, the primary focus of this study is to design effective agricultural insurance. Broadly speaking, agricultural insurance can be classified into two main types of design, namely indemnity-based and index-based. The key difference between them lies in how the indemnity payment is being determined. The payout from the indemnity-based insurance links directly to the agricultural producer's actual incurred loss while the payout from the index-based insurance depends explicitly on some pre-specified indexes. Plausible indexes include those based on weather (such as temperature and rainfall) or remote sensing satellite imagery (such as NDVI). From the point of view of the agricultural producers, indemnity-based insurance is preferred as it directly protects the actual incurred agricultural loss. The insurance providers, on the other hand, prefer index-based insurance as it entails fewer administrative and underwriting expenses. Moreover, index-based insurance also has the added advantage of alleviating moral hazard and anti-selection since its payout depends on a pre-defined index that is transparent and does not subject to manipulation, refer to Skees (1999), Martin et al. (2001), Turvey (2001), and Barnett and Mahul (2007). These advantages are particularly more important in developing countries where farms are typically small [such as 87% of the world's small farms (<2 ha) are in Asia] and hence underwriting indemnity-based insurance can be impractical. Studies by Hazell (1992) and Skees et al. (1999) alluded that the traditional indemnity-based approaches to crop insurance are not sustainable and the index-based insurance becomes a viable solution. See also Collier et al. (2009).

Despite the advantages of index-based insurance, it remains challenging to design index-based insurance that is effective and sustainable. The difficulty stems from the construction of the index. An inappropriate specification of an index can lead to unacceptable high basis risk. Here basis risk refers to the mismatch between the actual loss suffered by the producer and the indemnity from the insurance policy; this is triggered by the imperfect correlation between the producer's incurred loss and the chosen index, refer to, for example, Woodard and Garcia (2008), Elabed et al. (2013), Carter et al. (2015), and Conradt et al. (2015). Because of the imperfect correlation, it is possible to generate the following two types of errors in quantifying basis risk, commonly known as Type I and Type II. Type I error arises when a producer does not receive any indemnity despite there being an incurred loss while Type II error attributes to receiving indemnity even though there is no incurred loss to the producer. Both forms of errors are undesirable and raise concerns about the effectiveness of index-based insurance, refer to Woodard and Garcia (2008) and Norton et al. (2012) for additional discussion on quantifying basis risk.

The presence of basis risk is the key reason why the demand for index-based insurance has remained relatively low. For example, despite substantial premium subsidy (often in excess of 60%), index-based insurance piloted in Malawi and India (Gine, 2009; Cole et al., 2011) has not been very successful, with participation rates of only 20–30%. For this reason, designing index-based insurance that is beneficial to all stakeholders is an ongoing challenging problem. In the context of agriculture, weather risk is the dominant cause of agriculture loss, with some estimates that as much as 70–90% of crop loss is attributed to adverse weather (Olen and Auld, 2019). Hence, accurate modeling of crop yield and its relation to weather variables is an important first step in the design of weather-based index insurance (WII). There is quite an extensive literature that discusses the feasibility of WII for agriculture. The weather-yield models in many of these studies are based on a regression approach. For example, Thompson (1986, 2013) establish a multiple regression framework to explain the relationship between weather, technology, and crop production. Based on a large fine-scale weather dataset with county-level crop yields, Roberts and Schlenker (2011, 2012) explore the non-linear relationship between weather and crop yields and conclude that temperatures have different effects on plants during different phases. Roberts et al. (2012) propose a weather-yield regression model by exploiting a large fine-scale weather dataset and including the vapor pressure deficit factor as an explanatory variable. Extreme temperature indexes are shown to be more relevant to crop yield by agronomic experiments, and an econometric model linking yields to extreme weather index could make a prediction for the yield by regression equation (Schlenker et al., 2006; Schlenker and Roberts, 2009). Extreme temperatures, such as Growing Degree Days (GDD), Heating Degree Days (HDD), and Cooling Degree Days (CDD), have been the most popular indexes that focus on common weather derivatives (Mueller and Gradi, 2000; Turvey, 2001; Cao and Wei, 2004; Richards et al., 2004). Most of these works have been devoted to designing index-based weather insurance contracts by using the extreme weather index during the growing period (see Vedenov and Barnett, 2004; Woodard and Garcia, 2008). In hedging the risk of extreme temperature, many works set up new weather indexes and then use the regression model based on these indexes and yields for hedging crop yields (Manfredo and Richards, 2009; Xu et al., 2010; Yu and Babcock, 2010). See Odening and Shen (2014) for additional discussion on the challenges of hedging weather risk in agriculture.

Based on the lessons learned from the above studies, the purpose of the WII promotion should be to improve policy design and actuarial pricing confidence and to develop a generally applicable platform for various situations. Schlenker and Roberts (2009) indicate that crop scientists found the roughly optimal growing temperatures for corn (29°C), soybeans (30°C), and cotton (32°C), and the temperatures above the optimum were harmful to yield. However, no studies investigate the impact of employing optimal baseline temperatures on the WII contract design. The work of Schlenker and Roberts (2009) is expanded upon in this study by exploring the effect of optimal baseline temperatures on weather-yield models. This study develops a weather-yield model using the optimal approach and investigates whether this approach minimizes the basis risk and improves the efficiency of the designed WII policies.

In this study, we propose a new design of WII. We begin by offering a new weather-yield model that links crop yields to weather variables. Rather than modeling crop yields using the weather variables aggregated over the growing season, our proposed weather-yield model has the flexibility of incorporating weather variables monthly, thus reflecting that the effect of weather variables may be different depending on the growth stages. Two key features of our proposed regression models are (i) the optimal baseline temperatures in determining the weather indexes are obtained *via* an optimization model that minimizes the regression model's Root Mean Square Error (RMSE). The final adopted regression model provides the best-fit regression coefficients and gives the lowest RMSE among possible baseline temperatures. After estimating all possible baseline temperatures from all potential predictor variables, variables are selected based on the expected changes in RMSE. Here, a grid search with the Leave-One-Out Cross-Validation (LOOCV) is used to minimize the model's prediction RMSE to find the optimal parameter combination; (ii) in addition to using extremely high temperature as one of the explanatory variables, we also introduce a new weather variable which captures the extremely low temperature. In our context, minimizing the RMSE is a suitable objective as the RMSE is a yardstick for determining the quality of the regression model in that it quantifies the prediction power of the underlying model. Hence, constructing a WII using a weather-yield model with the lowest RMSE can reduce the WII's basis risk.

To illustrate our proposed regression model and demonstrate our proposed WII's effectiveness, we conducted an empirical study by assuming a contract portfolio in Anhui province, China, interested in WII hedging its middle-season rice production. We consider middle-season rice data in the Lujiang, He, Wangjiang, Tongcheng, Dangtu, Xiuning, Guichi, Dingyuan, Wuhu, and Feidong counties. These counties are major producers of middle-season rice in the Anhui province. Our interest in designing WII for rice is that China is the largest rice production and consumption country globally, producing and consuming about 30 percent of the world's rice (USDA, 2018). Hence designing an appropriate mitigation strategy for rice farmers is of paramount importance in China and is an active research area. For example, Yang et al. (2015) designed a particular weather index for the insurance of heat damage to rice in the Anhui province and compared the insurance compensation with the actual loss. Shi and Jiang (2016) propose a composite weather index insurance model for rice using a panel model and evaluating its efficiency in hedging the yield risk in Jiangsu province, China.

High temperatures and a poor water supply limit the growth of middle-season rice plants. Yang et al. (2015) studied yield reduction due to drought during different developmental phases in rice; they found that rice is susceptible to temperature during the phase between ‘Blooming' and ‘Grain filling.' Numerous WII contracts for rice have been developed in China. In 2009, the Guoyuan Agricultural insurance company issued the first rice WII contract in Changfeng county's Shuihu town. This WII contract includes the cumulative high-temperature difference index and the drought index. In the scenario that the cumulative high-temperature difference between July 30 and August 15 exceeds the high-temperature trigger, farmers would be compensated for high temperatures. In addition, farmers would be available for drought payments if the accumulated precipitation between May 15 and August 13 or September 1 and October 15 exceeds the drought trigger. In 2013, Guoyuan Agricultural Insurance Company provided a new WII contract to the farmers in Wuhu and Nanling county that considered only a single extreme heat index.

Existing WII contracts for rice in the Anhui province are designed using the high-temperature difference and accumulated rainfall over a specific period as indices. They do not consider indicators such as GDD, HDD, and CDD that are currently prevalent. In this study, we develop a hypothetical standard model using GDD and monthly average precipitation as benchmarks. Then we propose innovative approaches for enhancing the performance of this weather-yield model. Note that if the new approach can make the existing weather-yield model more accurate, this approach is superior to the existing one.

While we have used middle-season rice data to illustrate our methodology and the design of WII, it is important to note that our proposed methodology and optimization are very general; they can be applied to any crop (with an appropriate choice of weather index) to improve the weather-yield models.

## Data Source

**Figure 1:**
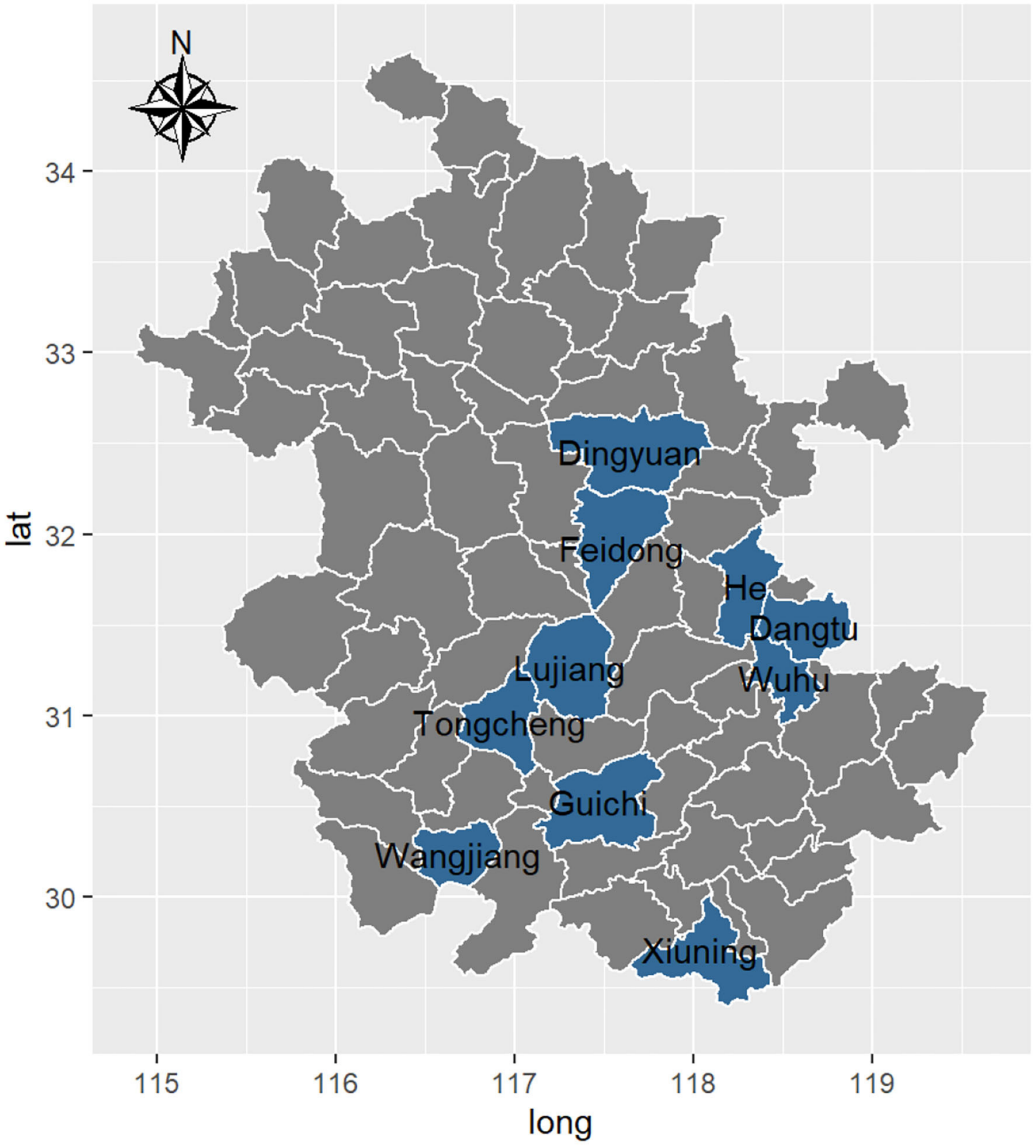
County boundaries in Anhui province.

This section provides details on the datasets. As seen in Figure 1, this study investigates mid-season rice yields in ten counties in the Chinese province of Anhui, including Lujiang, He, Wangjiang, Tongcheng, Dangtu, Xiuning, Guichi, Dingyuan, Wuhu, and Feidong. The average yields (kg per mu) of mid-season rice for these counties from 1980 to 2012 are provided by the Chinese Ministry of Agriculture. Each county's average yield is calculated by dividing the total crop yields by the total harvested area (mu).

This study's meteorological datasets were generated by linear interpolation of weather station data provided by the China Meteorological Data Service Centre. Our interpolation process uses the inverse distance weighting (IDW) method to generate the weather variable for each grid cell. The area-weighted average of each meteorological variable for the target county is calculated using each grid cell area. The monthly average precipitation extends from 1980 to 2012, and the daily average temperature includes from 1952 to 2013.

## Methodology

### Detrending Yield

Empirically, the crop yields over time, in general, exhibit an upward trend. This phenomenon, in part, is attributed to the advances in technology (such as better farming practices and enhanced climate-resilient seeds). For this reason, it is important to “detrend” the yield data before any modeling. A plausible way of capturing the trend is *via* a linear function of the following form (see Woodard and Garcia, 2008):


(1) $$\begin{array}{l} Y_{t}=\alpha_{0}+\alpha_{1}t+\;\epsilon_{t} \end{array}$$

In the above model, the observed rice yield *Y*_*t*_ (per mu) in year *t* is captured by two components: the deterministic component α_0_ + α_1_*t* and the random component ϵ_*t*_. The parameter α_0_ measures the central tendency of the yield while α_1_*t* reflects the linear time trend of the yield. The random component ϵ_*t*_ captures the residual variation due to other factors (such as natural disasters). In the empirical study to be presented in the next section, the optimal parameters α_0_ and α_1_ are determined *via* the best linear fit.

From the linear trend model (1), the equivalent time-*t* yield detrended to an arbitrary year t^*^, denoted by $Y_{t}^{\det},$ is calculated by


(2) $$\begin{array}{l} Y_{t}^{\det}=Y_{t}+\alpha_{1}\left(t^{*}-t\right) \end{array}$$

In our empirical illustration, t^*^ is set to 2012 for all the counties as this is the most recent year for which we have the data for our middle-season rice data for each of the counties.

### Weather Measures and Weather-Yield Models

Multiple regression-based statistical models are widely used as an alternative to agronomic process-based models in predicting crop yields. The general form of the regression model can be expressed (Lobell and Burke, 2010):


(3) $$\begin{array}{l} Y_{t}^{\det}=\beta_{0}+\beta_{1}X_{1,t}+\beta_{2}X_{2,t}+\cdot \cdot \cdot +\varepsilon_{t}, \end{array}$$

where $Y_{t}^{\det}$ represents the detrended crop yield in the year *t*, *X*_1,*t*_, *X*_2,*t*_, …, are the explanatory weather variables, and ε_*t*_ is the error term. The effectiveness of the above multiple regression model critically depends on the choices of the explanatory weather variables, which, in turn, depend on the type of crops, location, etc. While there is no consensus on what kind of weather variables are for the explanatory weather variables (see Zhu et al., 2019), weather indexes, such as GDD, HDD, and CDD, and precipitation are commonly adopted to the regression model.

In our proposed regression models, we not only take into consideration typical weather indexes such as GDD and precipitations, but also other weather indexes based on generalizations of HDD and CDD. Following Roberts et al. (2012), we now describe the weather indexes of relevance to this study. Let *T*_*d*_ be the average temperature on day *d*. *T*_*d*_ is often defined as the average of the day's maximum and the minimum temperatures of the day. Then the GDD on day *d* is defined as


(4) $$\begin{array}{l} GDD_{d}=\max\left[\min\left(T_{d},\;t_{upper}\right)-t_{lower},0\right] \end{array}$$

where *t*_*lower*_ and *t*_*upper*_, *t*_*lower*_ < *t*_*upper*_ correspond to the lower and upper baseline temperatures. While the GDD quantifies crop development, we also consider two other weather indexes that are detrimental to crop growing. These two weather indexes are denoted as the Extreme Heating Degree Days (EHDD) and the Extreme Cooling Degree Days (ECDD), and are defined, respectively, as


(5) $$\begin{array}{l} EHDD_{d}=\max\left(T_{d}-t_{upper},0\right) \end{array}$$

(6) $$\begin{array}{l} ECDD_{d}=\max\left(t_{cold}-T_{d},0\right) \end{array}$$

Both of these weather indexes capture the extremal temperatures on day *d*in that EHDD quantifies extreme heat while ECDD measures extreme coldness. From the daily measures of *GDD*_*d*_, *EHDD*_*d*_ and *ECDD*_*d*_, it is useful to construct their equivalent indexes but on a monthly basis. By denoting *AGDD*_*tj*_, *AHDD*_*tj*_, and *ACDD*_*tj*_ as, respectively, the accumulated GDD, EHDD, and ECDD, in *j-th* month of year *t*, then we have


(7) $$\begin{array}{l} AGDD_{tj}=\sum_{d\in monthj}GDD_{d} \end{array}$$

(8) $$\begin{array}{l} AHDD_{tj}=\sum_{d\in monthj}EHDD_{d} \end{array}$$

(9) $$\begin{array}{l} ACDD_{tj}=\sum_{d\in monthj}ECDD_{d} \end{array}$$

The summation sums over all the daily observations for the given month. It should be emphasized that *GDD*_*d*_, *EHDD*_*d*_, and *ECDD*_*d*_ are the daily indexes corresponding to the respective *j-th* month in year *t*.

Finally, we use the notation *P*_*tj*_ to denote the monthly average precipitation (in centimeters) in the *j*-th month of year *t*. Based on the above weather indexes, we consider the following three weather-yield models:


$$\begin{array}{l} \;Model\;I:\\ \;Y_{t}^{\det}=\beta_{0}+\sum_{j=4}^{9}\beta_{1j}\cdot P_{tj}+\sum_{j=4}^{9}\beta_{2j}\cdot AGDD_{tj}+\epsilon_{t}\\ \;=f\left(\beta_{0},\beta_{1j},\beta_{2j},P_{tj},AGDD_{tj}\;\right)\\ \;Model\;II:\\ \;Y_{t}^{\det}=f\left(\beta_{0},\beta_{1j},\beta_{2j},P_{tj},AGDD_{tj}\right)\\ \;+\sum_{j=7}^{8}\beta_{3j}\cdot \;ACDD_{tj}\\ \;=f\left(\beta_{0},\beta_{1j},\beta_{2j},\beta_{3j},P_{tj},AGDD_{tj},AHDD_{tj}\;\right)\\ Model\;III:\\ \;Y_{t}^{\det}=f\left(\beta_{0},\beta_{1j},\beta_{2j},P_{tj},AGDD_{tj}\right)\\ \;+\sum_{j=4}^{5}\beta_{4j}\cdot \;ACDD_{tj}\\ \;=f\left(\beta_{0},\beta_{1j},\beta_{2j},\beta_{4j},P_{tj},AGDD_{tj},ACDD_{tj}\;\right) \end{array}$$

Remarks on the above three regression models:

- The parameter β_0_ is the intercept and the parameters β_*i*·_, *i* = 1, …, 4 are the regression coefficients of the model. These coefficients will be optimally determined using the stepped regression approach.
- The growing season for the middle-season rice in the 10 counties is assumed to be from April to September; hence we are regressing the monthly average precipitation and the monthly accumulated GDD from April to September. For the AHDD weather index, we use the monthly data from July to August (motivated by the agronomy consideration). See Schauberger et al. (2017) for the importance of incorporating extreme high-temperature weather variables.
- Unlike Roberts et al. (2012), where the adopted weather indexes are the accumulated values over the entire growing season, our proposed models exploit the weather indexes on a monthly basis.
- Model III is distinct from Model II in that it employs alternate explanatory variables *ACDD*_*t*4_ and *ACDD*_*t*5_. Numerous studies have demonstrated that both extremely high and extremely low temperatures can have a negative impact on plant growth (Shimono et al., 2007; Chen and Chen, 2017). In particular, for agronomic reasons, we are only considering April and May for ACDD in our suggested Model III. The progressive effect of the extreme cooling temperature index can be explicitly analyzed by comparing Model III to Model I.

### Enhanced Weather-Yield Model *via* Minimization RMSE

One of the challenges of using Model I, Mode II, and Model III is that the models require as input the values of *t*_*lower*_ *and* *t*_*upper*_; these baseline values affect weather indexes GDD, EHDD, and ECDD. An inappropriate selection of these values can adversely affect the effectiveness of Model I, Model II, and Model III. These two parameter values are highly dependent on the location, climate, crop types, etc. See, for example, Yoshida et al. (1981) and Sanchez et al. (2014). Specifically, the second study suggests *t*_*lower*_= 20°C and *t*_*upper*_= 30°C. And crop scientists found the approximate optimal temperatures for corn (29°C), soybeans (30°C), and cotton (32°C) (Schlenker and Roberts, 2009).

The core concept of our proposed approach is to describe the task at hand as an optimization problem that determines *t*_*lower*_ and *t*_*upper*_ parameters optimally. The adopted criterion in our proposed optimization problem formulation is the RMSE. This criterion ensures that the standard deviation of the prediction errors of the underlying regression model is optimally minimized by selecting the values of *t*_*lower*_ and *t*_*upper*_ that minimize the RMSE. Consequently, our proposed method is based on historical data and is scientifically and statistically valid. It captures the predictive ability of the weather-yield model, indicating a superior model.

We will follow the steps below to obtain our final optimal weather-yield model calibrated to the middle-season rice data of the counties in Anhui province. Note that the predetermined set of weather indexes (i.e., explanatory variables) in the weather-yield Model I, Model II, and Model III require *t*_*lower*_ and *t*_*upper*_ as inputs. We determine the optimal values of temperature baselines for a given collection of model explanatory variables by solving the optimization problem. Third, to establish model parsimony and the ideal combination of explanatory variables, a stepwise regression approach and grid search with LOOCV are utilized.

We select the optimal combination of temperature parameters by iterating through all optional values of the temperature baseline. At each stepwise regression, the optimization problem is solved repeatedly in order to determine the optimal values of temperature parameters with the minimum attainable RMSE. And the temperature integer parameter range is set as *t*_*lower*_ ∈ [8, 21] and *t*_*upper*_ ∈ [30, 35] for the agronomy consideration (Yoshida et al., 1981; Schlenker and Roberts, 2009).

### Efficiency Analysis

Insurers offer the WII product because they believe it will reduce the economic implications of weather risk on farmers. This contract's efficacy is primarily determined by the basis risk and the farmers' evaluation of whether the WII contract can effectively protect their revenues.

We discuss a possible contract design for WII based on the regression models and then evaluate their relative effectiveness empirically for farmers aiming to hedge their crop production. The WII contract, designed in the style of the European put option, is suitable for weather-related loss indemnity. The WII contract allows the farmer to execute the option at a particular time. The owner will receive a payout if the expected yield from the weather-yield model is less than the triggered yield.

Let $I_{t}^{WII}$ denote the indemnity of a WII payable at the end of the contract year *t*. Then a plausible WII can be constructed from


(10) $$\begin{array}{l} I_{t}^{WII}=\max\left(K_{t}-\tilde{Y_{t}^{\det}},0\right)\cdot P \end{array}$$

where *K*_*t*_ is the triggered yield in year *t*, $\tilde{Y_{t}^{\det}}$ is the predicted yield per mu in year *t* determined from the calibrated weather-yield model, and *P* is the price election that corresponds to the crop's market price in the contract year *t*. At the inception of the contract, i.e., the beginning of year *t*, the triggered yield *K*_*t*_ is a known constant obtained from $K_{t}=\bar{Y}\times CL.$ Here, $\bar{Y}$ is the average historical yield and *CL* is the coverage level that captures the proportion of the historical yield the farmer wishes to insure. A farmer with the above WII policy has downside protection in the sense that his crop yield for the year will not fall below *K*_*t*_. If the crop yield at the end of the contract year of the WII (as dictated by $\tilde{Y_{t}^{\det}}\;$ from the adopted regression model) is less than *K*_*t*_, then the WII policy assumes that the farmer incurs a loss and an indemnity of the amount $\left(K_{t}-\tilde{Y_{t}^{\det}}\right)\cdot P$ is compensated to the farmer. On the other hand, if $\tilde{Y_{t}^{\det}}\;$ is greater than *K*_*t*_, then the contract assumes that there is no loss to the farmer so there is no payout from the WII contract. Thus, a WII contract with the above indemnity function (10) resembles a put option and the strike rate *K*_*t*_ becomes the minimum guaranteed crop yield for the farmer who has the WII policy.

Where *K*_*t*_ is the triggered yield in year t, $\tilde{Y_{t}^{\det}}$ is the predicted yield per mu in year t determined using the calibrated weather-yield model, and *P* is the price election corresponding to the crop's market price in the contract year *t*. At the inception of the contract, i.e., the beginning of year *t*, the triggered yield *K*_*t*_ is known constant from $K_{t}=\bar{Y}\times CL$. Here, $\bar{Y}$ represents the average historical yield, and *CL* is the coverage level representing the proportion of the average historical yield that the farmer intends to insure. A farmer with the above WII contract has downside protection in that his annual crop yield will not fall below *K*_*t*_. If the predicted crop yield in the contract year of the WII (as dictated by $\tilde{Y_{t}^{\det}}$ from the adopted regression model) is less than *K*_*t*_, then the WII policy considers that the farmer has suffered a loss and compensates them with an indemnity equal to $\left(K_{t}-\tilde{Y_{t}^{\det}}\right)\cdot P$. Alternatively, if $\tilde{Y_{t}^{\det}}$ is greater than *K*_*t*_, the contract assumes there is no loss to the farmer, resulting in no payment under the WII contract. Thus, a WII contract with the above indemnity function (10) resembles a put option and the trigger *K*_*t*_ becomes the minimum guaranteed crop yield for the WII policy-holding farmer.

Because the trigger *K*_*t*_ is based on the farmer's historical crop production, it can be interpreted as the crop yield the farmer expects to produce in year *t*. For this reason, *K*_*t*_ can serve as a yardstick for measuring the farmer's crop loss. More specifically, the farmer's actual crop loss can be determined from (10) by replacing $\tilde{Y_{t}^{\det}}$ with the farmer's realized crop yield $Y_{t}^{\det}$ for the year. We use $I_{t}^{actual}$ to denote the resulting actual loss that is incurred by the farmer in year *t*, then


(11) $$\begin{array}{l} I_{t}^{actual}=\max\left(K_{t}-Y_{t}^{\det},0\right)\cdot P \end{array}$$

Note that the indemnity $I_{t}^{WII}$ from the WII depends on $\tilde{Y_{t}^{\det}}$ (which, in turn, is prescribed by the adopted regression model) while $I_{t}^{actual}$ depends on the crop yield $Y_{t}^{\det}$ that is actually experienced by the farmer. Because both $I_{t}^{WII}$ and $I_{t}^{actual}$ depend on different underlying variables, it is not surprising that $I_{t}^{WII}$ and $I_{t}^{actual}$ need not match exactly. The discrepancy between $I_{t}^{WII}$ and $I_{t}^{actual}$ gives rise to the so-called basis risk. The effectiveness of a WII in serving as a hedge to a farmer, therefore, crucially depends on the severity of the basis risk.

To examine the efficacy of WII, we undertake the following empirical analysis. We assume a contract portfolio exists that hedges the mid-season rice yield for each of these counties (one mu per county). The contract portfolio's yields were assumed to match county-level yields. All farmers participating in WII will be paid if the predicted yield is less than the triggered yield. Actual farm-level yields may differ from county-level yields in quantity and variability, and WII's influence on risk exposure at an individual farm may differ from the county-level effect. Variability in actual farm yields would reduce WII's risk-reducing efficiency in practical applications compared to its performance for the representative contract portfolio (Vedenov and Barnett, 2004).

The WII is assumed to have a 100% coverage level, *P* = *1*, *K*_*t*_ is given by the average of the past historical yields, and the predicted yield $\tilde{Y_{t}^{\det}}$ is given by the weather-yield models, i.e., Model I, Model I-Optimal, Model II-Optimal, and Model III-Optimal.

The farmers are more concerned with the severity of the deviation of the actual loss relative to the WII's indemnity (i.e., $I_{t}^{actual}$ relative to $I_{t}^{WII}$). The first criterion for selecting the superior weather-yield modeling approach is to compare the basis risk fit by the various approaches. Because of the limited data availability (1980–2012 for counties in Anhui province), we establish an out-of-sample method for assessing the basis risk of the weather-yield models. First, the basis risk of the contract year 2005 is estimated separately based on the weather-yield models using weather and yield data from 1980 to 2004. Then, the basis risk of the contract year 2006 is estimated using the 1980–2005 datasets, and this process is repeated until the basis risk of the contract year 2012 is estimated using the 1980–2011 datasets.

Farmers are more concerned with the deviation between the actual loss and the WII's indemnity (i.e., $I_{t}^{actual}$ vs. $I_{t}^{WII}$). Comparing the basis risk fitted by the various weather-yield modeling approaches is the criterion for selecting the best approach. Due to the limited availability of data (1980-2012 for counties in the province of Anhui), we developed an out-of-sample method for evaluating the basis risk of the weather-yield models. Initially, the basis risk for the contract year 2005 is assessed individually using weather-yield models and data from 1980 to 2004. This approach is repeated until the basis risk of the contract year 2012 is evaluated using the 1980 to 2011 datasets.

The total basis risk associated with a group of contracts, indicated by Ω, is defined as follows:


(12) $$\begin{array}{l} BasisRisk_{\Omega }=\sum_{k\epsilon \Omega }|I_{t}^{WII}-I_{t}^{actual}| \end{array}$$

Where $I_{t}^{WII}$ is the estimated payment (in yuan per mu), and $I_{t}^{actual}$ is the actual loss for a contract in the coverage year *t*. From 2005 to 2012, we assessed the total basis risks of the sample portfolio in Anhui province. By comparing the results, we can identify which of these modeling approaches protects farmers against weather risk most effectively.

Additionally, the basis risks are classified as “false negative” or “false positive.” The former means insufficiently compensated losses for farmers when experiencing yield losses, while the latter means overpaid than the actual yield losses or even no yield losses. For insurance design, “false negative” is generally considered more important (Benami et al., 2021; Vroege et al., 2021). Hence, this study evaluates the efficacy of WII insurance in protecting farmers' income through the use of two additional indicators of basis risk:


(13) $$\begin{array}{l} BasisRisk_{\Omega }^{FP}=\sum_{k\epsilon \Omega }\max\left(I_{t}^{WII}-I_{t}^{actual},\;0\right) \end{array}$$

(14) $$\begin{array}{l} BasisRisk_{\Omega }^{FN}=\sum_{k\epsilon \Omega }|min\left(I_{t}^{WII}-I_{t}^{actual},\;0\right)| \end{array}$$

Where the “false positive” basis risks $BasisRisk_{\Omega }^{FP}$ are used to measure the expected payouts larger than the actual losses connected with the group of contracts, there will be adverse selection for farmers and wasteful losses for the insurance firm. Similarly, the “false negative” basis risks $BasisRisk_{\Omega }^{FN}$ are used to determine contract payouts smaller than actual losses, meaning that farmers will not receive appropriate coverage even if they enroll in this WII contract.

The second criterion for selecting the superior weather-yield modeling approach is whether getting a WII policy can effectively protect the farmer's revenue. To determine whether WII policies based on Model I, Model I-Optimal, Model II-Optimal, and Model III-Optimal effectively guarantee policyholders' income. We evaluated the ability of the WII policy to reduce weather risk by comparing the income risk of farmers who obtained WII contracts to those who did not. The mean root square loss (MRSL) was used to quantify farmers' revenue risk exposure variation. This criterion has been utilized in various studies on agricultural commodity hedging (see Turvey and Nayak, 2003; Vedenov and Barnett, 2004; Kim et al., 2010). The following equation can be used to calculate the revenues of farmers who did not buy a WII contract:


(15) $$\begin{array}{l} R_{t}^{WO}=P\;*\;Y_{t}^{\det}, \end{array}$$

While the revenue of farmers who owned a WII contract was calculated as:


(16) $$\begin{array}{l} R_{t}^{W}=P\;*\;Y_{t}^{\det}+I_{t}^{WII}-\Gamma , \end{array}$$

Where P is the corresponding rice price, and Γ is the premium for the farmer.

The MRSL is a simple function of the semi-variance and is calculated for the revenues of farmers without and with the WII contract as follows:


(17) $$\begin{array}{l} MRSL_{without}=\sqrt{\frac{1}{T}\sum_{t=1}^{T}{\left[\max\left(P\cdot \bar{Y}-R_{t}^{WO},0\right)\right]}^{2}}, \end{array}$$

(18) $$\begin{array}{l} MRSL_{with}=\sqrt{\frac{1}{T}\sum_{t=1}^{T}{\left[\max\left(P\cdot \bar{Y}-R_{t}^{W},0\right)\right]}^{2}}, \end{array}$$

Where $\bar{Y}$ is the long-term average (target) yield, the actuarially fair premium **Γ** equals the expected payout. Using empirical rates, we approximate the actuarial premiums here. Specifically, the actuarial premium is determined by averaging historical contract payouts over the study period. Throughout the study period, a constant actuarial premium is applied to each county.

## Results and Discussion

Using middle-season rice data from the 10 counties in Anhui province, Table 1 depicts the optimal values of *t*_*lower*_ *and* *t*_*upper*_, along with the minimal RMSE. These values are obtained by applying the optimal algorithms to Model I, Model II, and Model III and with the additional integer constraints 8 ≤ *t*_*lower*_ ≤ 21, and 30 ≤ *t*_*upper*_ ≤ 35, to ensure *t*_*lower*_ < *t*_*upper*_. The initial conclusion that can be derived from Table 1 is that all three optimization models provide a lower RMSE than Model I ($t_{lower}=20^{o}C$ and $t_{upper}=30^{o}C$). Second, compared to $t_{lower}=20^{o}C$ and $t_{upper}=30^{o}C$, which has been proposed by other researchers, the optimal values of the baseline temperature derived from our proposed optimization framework are somewhat different. Lastly, an optimization weather-yield model that includes ACDD or AHDD in addition to the AGDD index will have a lower RMSE and better model fit than an optimization model that solely includes AGDD.

**Table 1:** The optimal values of *t*_*lower*_, *t*_*upper*_ and the RMSE for Model I, Model II, and Model III.

**Model**	**t_lower_**	**t_upper_**	**RMSE**
Model I	20°C	30°C	65.5899
Model I-optimal	8°C	34°C	63.782
Model II-optimal	9°C	34°C	62.4879
Model III-optimal	19°C	30°C	61.4811

*The $t_{lower}=20^{o}C$ and $t_{upper}=30^{o}C$ of Model I have been suggested by Yoshida et al. (1981)*.

The regression results from fitting the weather-yield models to the county-level middle-season rice yield data are summarized in Tables 2–**5**. Tables 3–**5** give the best fit regression results for Model I, Model II, and Model III in conjunction with the optimal baseline temperatures obtained from the optimization algorithm. We label these results as Model I-Optimal, Model II-Optimal, and Model III-Optimal, respectively. To benchmark against using the optimal baseline temperature, the results for Table 2 correspond to Model I but assume the suggested baseline temperatures *t*_*l*_=20°C and *t*_*u*_=30°C.

**Table 2:** The simulation results are based on Model I.

	**April**	**May**	**June**	**July**	**August**	**September**
**Index**						
Precipitation	/	−0.1280 (0.0767)	−0.0952 (0.0402)	−0.1841 (0.0499)	/	/
AGDD	0.6397 (0.4494)	−0.4568 (0.1762)	−0.2891 (0.1574)	−0.2820 (0.1916)	0.3994 (0.1496)	/

*(1) The final model gives the regression coefficients of the dummy variables for the counties of Dingyuan, Lujiang, Wuhu, and Xiuning by using other counties as a reference*.

*(2) The RMSE of the training model is 65.5899*.

*(3) The redundant weather indices are indicated by “/”*.

*(4) Yield is measured in the unit of kg/mu, where 1 mu = 1/15 hectare*.

*(5) Precipitation is measured in 1 mm, temperature in 1°C*.

**Table 3:** The simulation results are based on Model I-optimal.

	**April**	**May**	**June**	**July**	**August**	**September**
**Index**						
Precipitation	/	−0.187 (0.0816)	−0.1196 (0.0403)	−0.1883 (0.0484)	/	−0.1384 (0.0945)
AGDD	0.2210 (0.1226)	−0.5358 (0.1278)	−0.2952 (0.1512)	−0.2791 (0.1455)	0.4104 (0.1332)	/

*(1) The final model gives the regression coefficients of the dummy variables for the counties of Dingyuan, Feidong, Lujiang, Wuhu, and Xiuning by using other counties as a reference*.

*(2) The RMSE of the training model is 63.782*.

*(3) The redundant weather indices are indicated by “/”*.

*(4) Yield is measured in the unit of kg/mu, where 1 mu = 1/15 hectare*.

*(5) The precipitation is measured in 1 mm, and the temperature is measured in 1°C*.

First, note that the results in Tables 2–**5** show that not all monthly indexes over the growing season are significant; the redundant weather indices are indicated by “/.” This is a consequence of applying the stepwise regression method to eliminate some non-significant explanatory variables. This analysis suggests that it may be inappropriate to aggregate the weather indexes over the entire growing season as the effect of weather indexes on crop yields depends critically on the growth cycle of crops.

We now assess the impact of the regression models based on the proposed optimally determined baseline temperatures. Recall that the results of Model I in Table 2 are obtained using the baseline temperature *t*_*l*_=20°C and *t*_*u*_=30°C. The results of Model I-Optimal in Table 3 are also based on the same weather-yield model (i.e., Model I), except it uses the optimal baseline temperatures *t*_*lower*_ *and* *t*_*upper*_ that minimize the RMSE. Hence, comparing the Model I to Model I-Optimal results allows us to evaluate the incremental impact attributing to the optimal baseline temperatures. For the direct implementation of Model I, we have RMSE = 65.5899 for all the counties. By incorporating the optimal baseline temperature, the corresponding RMSE decreased to 63.782. The decrease in the RMSE signifies the effectiveness of our proposed optimization framework of seeking optimal baseline temperatures.

As shown in Table 4, when additional heating temperature explanatory variables (i.e., AHDD) are included in Model II, the regression results indicate that August AHDD is significant, and the RMSE statistic decreases. Model II-optimal reduces the RMSE from 63.782 to 62.4879 compared to Model I-Optimal. According to Table 5, Model III is more innovative in including additional cooling temperature explanatory variables (i.e., ACDD). The fitted regression results reveal that both the April and May ACDD are significant for the regression results, and there is a decrease in the RMSE statistic. Model III-Optimal reduces the RMSE from 63.782 to 61.4811 compared to Model I-optimal.

**Table 4:** The simulation results are based on Model II-Optimal.

	**April**	**May**	**June**	**July**	**August**	**September**
**Index**						
Precipitation	/	−0.1750 (0.0789)	−0.1135 (0.0392)	−0.1540 (0.0483)	/	−0.1511 (0.0929)
AGDD	0.1841 (0.1208)	−0.5427 (0.1250)	−0.2689 (0.1483)	−0.2229 (0.1430)	0.4275 (0.1307)	/
AHDD	NA	NA	NA	/	−181.7113 (49.2884)	NA

*(1) The final model gives the regression coefficients of the dummy variables for the counties of Dingyuan, Lujiang, Wuhu, and Xiuning by using other counties as a reference*.

*(2) The RMSE of the training model is 62.4879*.

*(3) The redundant weather indices are indicated by “/”*.

*(4) Yield is measured in the unit of kg/mu, where 1 mu = 1/15 hectare*.

*(5) The precipitation is measured in 1 mm, and the temperature is measured in 1°C*.

**Table 5:** The simulation results are based on Model III-optimal.

	**April**	**May**	**June**	**July**	**August**	**September**
**Index**						
Precipitation	/	−0.2234 (0.0785)	−0.1421 (0.0421)	−0.2659 (0.0498)	/	−0.1804 (0.0927)
AGDD	/	/	/	−0.6410 (0.1761)	0.5491 (0.1453)	0.3410 (0.1452)
ACDD	−0.3000 (0.1615)	2.4257 (0.4471)	NA	NA	NA	NA

*(1) The final model gives the regression coefficients of the dummy variables for the counties of Dingyuan, Feidong, He, Lujiang, and Xiuning by using other counties as a reference*.

*(2) The RMSE of the training model is 61.4811*.

*(3) The redundant weather indices are indicated by “/”*.

*(4) Yield is measured in the unit of kg/mu, where 1 mu = 1/15 hectare*.

*(5) The precipitation is measured in 1 mm, and the temperature is measured in 1°C*.

For Model I, Model I-Optimal, Model II-Optimal, and Model III-Optimal implementations, the RMSE values are 65.5899, 63.782, 62.4879, and 61.4811. These results indicate a significant advantage for the weather-yield model employing the optimal baseline temperatures and we can further improve the model's fitted results by using the heating and cooling temperature weather index.

From Table 6, in comparison to Model I, the weather-yield models based on the optimal procedure significantly reduce the Mean Squared Error (MSE) between predicted and actual yields, hence reducing the exposure risk in the design of the WII policy. Table 6 provides a quantitative assessment of the basis risk. Farmers are concerned about the severity of the difference between the actual loss and the WII's payment (i.e., $I_{t}^{actual}$ relative to $I_{t}^{WII}$). The relative efficacy of the WII derived from the optimal weather-yield model is presented with clarity. Using Model I, Model I-Optimal, Model II-Optimal, and Model III-Optimal, the basis risk of the representative contract portfolio is calculated to be 1,956.028, 1,884.49, 1,839.923, and 1,725.655 yuan. Using Model I as the benchmark, this results in 3.66% less basis risk for Model I-Optimal, 5.94% less basis risk for Model II-Optimal, and 11.78% less basis risk for Model III-Optimal.

**Table 6:** The basis risk of the representative contract portfolio.

	**Model I**	**Model I-optimal**	**Model II-optimal**	**Model III-optimal**
MSE	255,255.4	203,588.8	217,972.1	203,682.8
Basis risk	1,956.028	1,884.49	1,839.923	1,725.655

*We assume a single contract covering one mu of crop yield in each county, and the basis risk is the total basis risk of all contracts for these ten counties from 2005 to 2012. The unit of basis risk is yuan. The total actual yields across all counties are 33,946.53 kg, therefore, the basis risk proportion of the actual yields is 5.76, 5.55, 5.42, and 5.08%, respectively*.

Table 7 compares the “false negative” and “false positive” basis risk associated with the WII contract using weather-yield models developed with the optimum approach. The risk of expected payouts exceeding actual losses can be quantified using the “false positive” basis risk. The lower the “false positive” basis risk, the less likely the farmer will get a WII contract payment that exceeds the actual loss. Using Model I, Model I-Optimal, Model II-Optimal, and Model III-Optimal, the portfolio's “false positive” basis risk is 1,044.278, 946.8404, 891.9111, and 974.4557 yuan for the typical contract portfolio. Using the “false negative” basis risk as a measurement, one can also determine the likelihood that the expected payout would be less than the actual losses. The lower the risk of a “false negative” basis risk, the less probable it is that farmers will incur losses greater than the amount of the policy payout. The “false negative” basis risk for the representative contract portfolio is 911.7496, 937.6493, 948.0115, and 751.1995 yuan when employing Model I, Model I-Optimal, Model II-Optimal, and Model III-Optimal, respectively. Using Model I as a benchmark, it is clear that Model II-Optimal could reduce the “false positive” basis risk most among all the models by 14.59%, and that Model III-Optimal could reduce the “false negative” basis risk most among all the models by 17.61%.

**Table 7:** The “false positive” and “false negative” basis risk of the representative contract portfolio.

	**Model I**	**Model I-optimal**	**Model II-optimal**	**Model III-optimal**
“False positive” basis risk	1,044.278	946.8404	891.9111	974.4557
“False negative” basis risk	911.7496	937.6493	948.0115	751.1995

*We assume a single contract covering one mu of crop yield in each county, and the basis risk is the total basis risk of all contracts for these ten counties from 2005 to 2012. The unit of basis risk is yuan*.

In this case, the most reducing basis risk WII contract is produced using the optimal algorithm. This indicates that the optimal weather-yield models are superior and that the impact of including the extreme cooling weather index in the weather-yield model is not negligible.

Lastly, we use MRSL to evaluate the losses of farm revenue in extreme weather when the WII contract is taken vs. when it is not, as well as the difference between the revenue risk of purchasing a WII contract based on a different modeling approach. Table 8 depicts the measurement of the reduction in revenue risk for farmers from purchasing WII contracts based on different models. This evaluation is based on the expected payout of the selected contract portfolio and the actuarially fair premiums. Since the government subsidizes the premiums of farmers who purchase agricultural insurance, the revenue risk of farmers is calculated under the hypothesis that the actual premiums paid are 100 percent, 50 percent, and 30 percent of the actuarial premiums, respectively. When farmers pay <50% of the actuarial premium, the WII contract based on Model I, Model I-Optimal, Model II-Optimal, and Model III-Optimal effectively reduces revenue risk and protects farmers' income. It is important to highlight that only the WII contract based on Model III-Optimal can reduce the revenue risk of farmers when the actual premium is paid in full.

**Table 8:** The efficiency of the WII contract as Measured by Mean Root Square Loss (MRSL).

**Actual premium**		**Out-of-sample (2005–2012)**		
		**Without contract**	**With contract**	**Percent change**
100% fair premium	Model I	266.8971	283.2946	6.14%
	Model I-optimal		286.8537	7.48%
	Model II-optimal		283.5939	6.26%
	Model III-optimal		231.4057	−13.30%
50% fair premium	Model I	266.8971	256.207	−4%
	Model I-optimal		260.3386	−2.46%
	Model II-optimal		260.0747	−2.56%
	Model III-optimal		207.2157	−22.36%
30% fair premium	Model I	266.8971	245.6895	−7.95%
	Model I-optimal		250.241	−6.24%
	Model II-optimal		251.383	−5.81%
	Model III-optimal		198.1609	−25.75%

*We assume a single contract covering one mu of crop yield in each county, and the MRSL for these ten counties from 2005 to 2012 is the sum of the MRSL for all contracts. The unit of measure is the yuan*.

In addition, compared to Model I, Model I-Optimal, and Model II-Optimal, the WII contract based on Model III-Optimal significantly reduces the revenue risk for farmers. In contrast, Model I-Optimal, and Model II-Optimal cannot significantly reduce income risk compared to Model I. It indicates that Model III-Optimal, which incorporates the extreme cooling weather index, is much more effective at reducing risk. This model can reduce revenue risk without government subsidies, whereas WII contracts employing other models can only reduce income risk for farmers when government subsidies are provided.

## Conclusion

This study proposed weather-yield models relating weather indexes to crop yields. The proposed weather-yield models were more flexible in that they could capture the month-to-month variations of the weather indexes and reflect the effect attributed to the extreme cooling weather and the extreme heating weather. A stepwise regression method coupled with a new optimization approach was proposed to calibrate the crop yield data. We demonstrated the impact of our proposed modeling approach by using the middle-season rice data in the counties of Anhui province. Some notable empirical findings were:

- Using our proposed optimization approach of maximizing the adjusted R-squared, the optimal baseline temperatures were model-dependent. These values differed from the suggested values of *t*_*l*_ = 20°C and *t*_*u*_ = 30°C (see Yoshida et al., 1981).
- The impact of optimal baseline temperatures was highlighted in the RMSE of the model. We observed a remarkable decrease in RMSE for weather-yield models by using the optimal baseline temperatures.
- Contrasting Model II-Optimal with Model III-Optimal indicated the importance of the extreme heat weather index (AHDD) and extreme cold weather index (ACDD) to crop yield modeling.
- Finally, we concluded a significant improvement in efficiency from the hypothetical WII contracts constructed based on our proposed optimal regression models.

In conclusion, our extensive empirical studies demonstrated the effectiveness of our proposed optimal regression models and the resulting optimal design of WII. It should be emphasized that while we had resorted to the middle-season rice data in the counties of Anhui province as illustrative examples, the proposed optimal approach was very general. Similar regression models and similar optimization ideas could be applied to other crops to determine the optimal baseline temperature. This work can avoid arbitrary assigning baseline temperatures while we could expect similar efficiency gain.

## Data Availability Statement

All data used in this study are publicly available. The processed data needed to reproduce this study are available at Agrisk-tools (http://www.agrisk-bigdata.com/site/data-query).

## Author Contributions

YS conceived and designed the study, provided the idea, performed the data analysis, analyzed the results, and wrote the manuscript.

## Conflict of Interest

The author declares that the research was conducted in the absence of any commercial or financial relationships that could be construed as a potential conflict of interest.

## Publisher's Note

All claims expressed in this article are solely those of the authors and do not necessarily represent those of their affiliated organizations, or those of the publisher, the editors and the reviewers. Any product that may be evaluated in this article, or claim that may be made by its manufacturer, is not guaranteed or endorsed by the publisher.
